# Protective Effects of Combined Intervention with Adenovirus Vector Mediated IL-10 and IGF-1 Genes on Endogenous Islet β Cells in Nonobese Diabetes Mice with Onset of Type 1 Diabetes Mellitus

**DOI:** 10.1371/journal.pone.0092616

**Published:** 2014-03-24

**Authors:** Lijuan Zhang, Yanyan Chen, Cheng Li, Xiaojie Lin, Xiaoli Cheng, Tang Li

**Affiliations:** 1 Department of Pediatrics, Affiliated Hospital of Medical College Qingdao University, Qingdao, Shandong, China; 2 Department of Pediatrics, People’s Hospital of RiZhao, Rizhao, Shandong, China; 3 Department of Medical Center, Qingdao Sanatorium of Shandong Province, Qingdao, Shandong, China; Children’s Hospital Boston/Harvard Medical School, United States of America

## Abstract

**Objective:**

To investigate the protective effects of combined intervention with adenovirus vector mediated interleukin 10 (IL-10) and insulin-like growth factor 1 (IGF-1) genes on islet β cells in nonobese diabetes (NOD) mice with type 1 diabetes mellitus (T1D) at early stage.

**Methods:**

Twenty-four female NOD mice at onset of diabetes and aged 17–20 weeks old were randomly divided into four groups. Mouse 1, 2 and 3 groups were intraperitoneally injected 0.1 ml of Ad-mIGF-1, Ad-mIL-10, and combined Ad-mIGF-1 and Ad-mIL-10, respectively. Mouse 4 group were used as diabetes control. In addition, six age- and sex-matched non-diabetic NOD mice were intraperitoneally injected 0.1 ml of PBS and assigned 5 group as normal controls. All mice were weekly monitored for body weight, urine glucose and blood glycose, and sacrificed 3 weeks after injection. Their serum levels of IL-10, IGF-1, IFN-γ, IL-4 and C-peptide were measured and the degree of insulitis and the local expression of IGF-1 and IL-10 gene were observed.

**Results:**

1) IL-10 and IGF-1 levels in serum and pancreas were enhanced in 1, 2, and 3 groups; 2) serum INF-γ level was decreased while serum IL-10 and IL-4 levels were increased in 1, 2 and 3 groups, and these alterations were more significant in 3 group than 1 and 2 groups (*P<*0.01); 3) C-peptide level was not enhanced in 1 group, but significantly increased in 2 and 3 groups, and these increases were more significant in the latter (*P<*0.01); 4) Three weeks later, the body mass of mice in 2 and 3 groups decreased significantly (*P<*0.05).

**Conclusion:**

The administration of adenovirus vector mediated IL-10 and/or IGF-1 gene showed limited immune regulatory and protective effects on islet β-cells in NOD mice with T1D at early stage, and no significant reduction in insulitis, blood glucose and body weight.

## Introduction

Type 1 diabetes mellitus (T1D) is an autoimmune disease characterized by insufficient insulin secretion and progressive damage of islet β-cells [1], [2]. Currently, its incidence is increasing worldwide in children [3]. T1D is caused by the interactions between genetic predisposition [4], [5] and environmental factors [6], [7], . It has been demonstrated to be a T cell-mediated disease. A current hypothesis is that B-cell also play an immportant role in the development of T1D by regulate T-cells [10]. The balance between Th1 and Th2 cells appears to be vitally important. Hence, a shift of the immune systen from Th1-like immunity to Th2-like immunity may represent an attractice and reasonable therapeutic strategy for T1D. Some studies have been reported that there are some methods were effective in preventing T1D but ineffective in reversing T1D [11], [12], [13]. Th2 cytokines (in particular IL-10), which could inhibit the function of Th1 cells and activity of Th1 cytokines, are considered as effective therapeutic factors for T1D [14], [15].

IL-10 was first described as cytokine synthesis inhibitory factor(CSIF) [16]. We have previously found that combined with transgenic technology, insulin-secreting cells that overexpress IL-10 by in vitro infection of Ad-rIL-10 still have insulin secretion function even at high glucose condition [17]. In addition, IL-10 over-expression can inhibit IL-1β-induced Fas expression and apoptosis of insulin secreting cells [18], reduce the incidence of diabetes of prediabetic NOD mice, and delay or even prevent T1D development. Islet β-cells have ability to regenerate themselves [19], [20], which mainly dependents on insulin-like growth factor-1 (IGF-1) [21], [22]. IGF-1 has insulin-like metabolic effects. Our previous studies have found that IGF-1 acts on insulin-secreting cells [23], [24], [25], NOD mice [26] and diabetic rats [27] in vivo, reducing islet inflammation, promoting cell proliferation, and inhibiting against apoptosis. The key for T1D treatment is to recover the proliferation and function of endogenous islet β-cells and to prevent autoimmunity that could damage islet β-cells. In this study, we conducted combined intervention of IL-10 and IGF-1 in NOD mice with diabetes at onset stage and evaluated its protective effects on the residual islet β- cells, hoping to provide a theoretical basis for early clinical treatment of T1D.

## Materials and Methods

### Ethics

This study was carried out in strict accordance with the Guide for the Care and Use of Laboratory Animals issued by the National Institutes of Health. The protocol was approved by the Committee on the Ethics of Animal Experiments of Affiliated Hospital of Medical College Qingdao University. Every effort was made to minimize the animals’ suffering. The use of the animals in this study was approved by the Qingdao University Institutional Animal Care and Use Committee.

### Animals and Treatment Protocol

Female NOD mice at age of 7–9 weeks old and weight 19∼21 g were purchased from the Chinese Academy of Medical Sciences Institute of Experimental Animals and have been maintained at the Animal Center of the Affiliated Hospital of Medical College Qingdao University. All the mice were kept in specific pathogen-free conditions in a 12-h dark/light cycle housed in ventilated filter cages with free drinking and eating. These mice had 80% natural incidence of diabetes at age of 30 weeks old [28], [29]. Human embryonic kidney 293 cells were kindly provided by Professor Bing Luo of Medical College Qingdao University and cultured to amplify adenoviruses. Ad-mIL-10 and Ad-mIGF-1 with titer of 1.0×10^9^ pfu/mL were kindly provided by Professor Zhihong Chen of the Department of Pediatrics, the Affiliated Hospital of Medical College Qingdao University.

24 female NOD mice at age of 17–20 weeks old and with diabetes diagnosed within 3 days were randomly divided into 4 groups. At the onset stage of T1D, mice in groups 1, 2 and 3 were intraperitoneally injected 0.1 ml of Ad-mIGF-1, Ad-mIL-10, and combined Ad-mIGF-1 and Ad-mIL-10, respectively. Mice in group 4 were used as diabetes control. In addition, six age and sex matched nondiabetic NOD mice were intraperitoneally injected 0.1 ml of PBS and assigned in group 5 as normal control.

Three weeks after treatment, all mice were sacrificed and their whole blood was collected after giving chloral hydrate. Serum specimens were collected by centrifugation for 15 min at 3000 r/min and stored at −80°C. To avoid repeated freeze and thaw, pancreas were separated immediately, formalin-fixed, embedded in paraffin and sliced. Specimens then stained with HE to observe islet inflammatory infiltration microscopically and immunohistochemical stained for IL-10 and IGF-1 to observe their local expression in pancreas.

### Monitoring for Diabetes

All mice were observed for polyphagia, polydipsia, polyuria, hair gloss and decreased vitalities, etc. Their body weight, urine glucose and blood glucose were measured weekly. Mice with glucosuria were tested for the blood glucose level and those showing >250 mg/dl of blood glucose two consecutive reading in a week were considered diabetic [30].

### Insulitis Score

Pancreas specimens stained with HE were examined using light microscope. Islet pathology of inflammatory infiltration was double-blind assessed microscopically by professional pathologists in 5 consecutive fields at 400×magnification and graded based on the following grading scale [31]: 0, no insulitis; 1, insulitis affecting <25% of the islet; 2, insulitis affecting 25–75% of the islet; 3, >75% islet infiltration. The number of the islets at different grades were counted and statistically analyzed.

### Immunohistochemistry

The expression of IL-10 and IGF-1 in pancreatic specimens were examined using immunohistochemical SABC method. Cells showing brownish yellow granules in the cytoplasm were considered as positive. The percentage of positive cells and staining intensity were calculated and used to quantity the expression of IL-10 and IGF-1. Specimens with <1%, 1∼10%, 11∼50%, 51∼80% and >80% of positive cells were scored as 0, 1, 2, 3, and 4, respectively. Specimens with staining intensity of light yellow, yellow, and brown particles were scored as 1, 2, and 3, respectively. The total immunohistochemical score of a specimen was calculated as the score for percentage of positive cells times the score for staining intensity, and expressed as (−), (+), (++) and (+++) for score 0, 1∼4, 5∼8, and 9∼12, respectively.

### Enzyme-linked Immunosorbent Assay (ELISA) for Serum C-peptide, IL-10, IL-4, IFN-γ and IGF-1 Levels

Serum C-peptide level may reflect the insulin secreting ability of islet β-cells and examed using enzyme-linked immunosorbent assay (ELISA) kit following the protocol provided by the manufacturer (R &D Inc., USA).

Serum IL-10, IL-4, IGF-1 and IFN-γ levels were measured using ELISA kits following the protocols provided by the manufacturer (R & D Inc. USA).

### Statistics

Data were shown as *±s* and analyzed for normality and homogeneity of variance. Differences between data showing normality and homogeneity of variance in multiple groups were compared using ANOVA and LSD test. Data lacking normality and showing heterogeneity of variance were analyzed using Log/Ln/Sin/Sqrt conversion followed by normality and homogeneity of variance. Those data that still failed to meet normality and homogeneity of variance were further analyzed using Games-Howell test and Kruskal-Wallis rank sum test. Data used for severity grading were analyzed using Kruskal-Wallis rank sum test. All statistical analyses were conducted using SPSS17.0 software and *P*<0.05 was considered statistically significant.

## Results

### Blood Glucose and Body Weight

Compared with mice in group 5, diabetic mice ate more food, drank more water and purged more urine. In addition, they were restless and easy to be irritated. The glossness of their body hair decreased. The above phenomena were more apparent 2 weeks after the onset of the disease in mice in group 4. The body weight of mice in groups 1, 2 and 3 at onset stage of the disease and within 3 weeks of injection showed no significant difference (*P*>0.05). However, after 3 weeks of injection, mice in groups 2 and 3 showed significantly lower body weight than mice in groups 4 and 1 (*P*<0.05). In addition, the body weight of mice in group 4 was not significantly different from that of mice in groups 1, 2 and 3 (*P*>0.05) (Table 1).

**Table 1:** Comparison of body weight of mice in different groups (*±s*, g).

Group	Body weight at onset of TM1D	Body weight after the onset of TM1D		
		One week	Two weeks	Three weeks
1	25.35±1.82	25.14±2.67	25.71±2.36	26.68±2.44
2	24.24±2.79	24.53±2.64	24.15±3.61	22.86±1.87*
3	22.97±1.34	23.43±2.03	24.08±1.73	23.12±1.73*
4	23.15±1.73	23.99±2.07	24.71±1.65	24.32±2.14
5	23.90±2.27	24.87±2.99	25.54±3.27	26.05±3.10

Notes: *P*<0.05 compared with groups 1 and 2.

**Figure 1:**
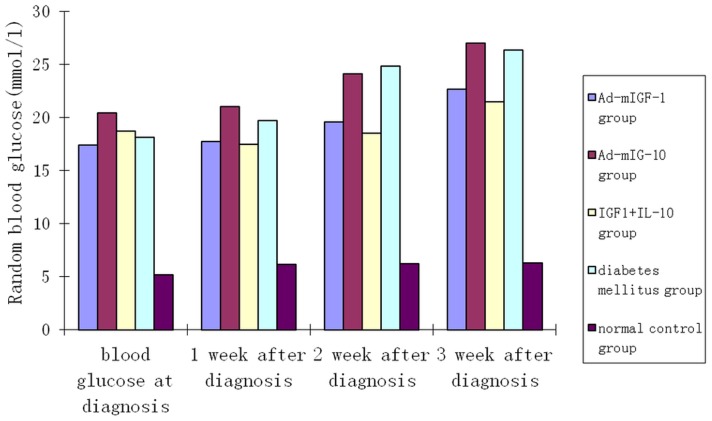
Comparison of serum glucose level of mice in different groups.

Before injection, the average random blood glucose of mice in group 4 was 5.17±1.56 mmol/L, which was similar to that (17∼21 mmol/L) of mice in other groups (*P*>0.05). After injection, the average random blood glucose of mice in group 3 was lower than that of mice in groups 1, 2, and 4 for three weeks, but higher than that of mice in group 5. But these differences were not statistically significant (*P*>0.05). Similarly, there were no significant difference in average random blood glucose among mice in groups 1, 2, and 4 (*P*>0.05) (Figure 1).

### Determination of Serum Cytokines

The levels of serum C-peptide, IFN-γ, IL-4, IL-10 and IGF-1 in each group were measured by ELISA and compared. As shown in Table 2, serum C-peptide level of mice in group 3 was significantly higher than that of mice in groups 1, 2, and 4, but significantly lower than that in group 5 (*P*<0.01). In addition, serum C-peptide level of mice in group 2 was significantly higher than that of mice in groups 1 and 4 (*P*<0.01), but there was no significant difference in serum C-peptide level between mice in groups 1 and 4 (*P*>0.05). Serum IFN-γ level of mice in groups 1, 2, 3 and 4 was significantly elevated compared with that of mice in group 5 (*P*<0.01). By contrast, serum IL-4 level of mice in groups 1, 2, 3, and 4 was significantly lower than that of mice in group 5 (*P*<0.01). In addition, compared with that of mice in group 4, serum IFN-γ level of mice in groups 1, 2 and 3 was significantly decreased, while serum IL-4 level was significantly increased (*P*<0.01). Moreover, serum IFN-γ level of mice in group 2 was significantly decreased compared with that of mice in group 1 (*P*<0.01), while there was no significant difference in serum IL-4 level between mice in these two groups. Serum IL-10 level of mice in groups 2 and 3 was significantly higher than that of mice in groups 1 and 4 (*P*<0.01). In addition, serum IL-10 level of mice in group 1 was significantly lower than that of mice in groups 2 and 3 (*P*<0.01), and there was no significant difference in serum IL-10 level between mice in groups 3 and 5 (*P*>0.05). Serum IGF-1 level of mice in groups 1 and 3 was significantly higher than that of mice in groups 2 and 4 (*P*<0.01), but significantly lower than that of mice in group 5 (*P*<0.01). In addition, serum IGF level of mice in group 2 was significantly higher than that of mice in group 4 (*P*<0.01).

**Table 2:** Comparison of levels of serum C-peptide, IFN-γ, IL-4, IL-10 and IGF-1(*x¯±s*).

Groups	C-peptide (ng/mL)	IFN-γ (pg/mL)	IL-4 (pg/mL)	IL-10 (pg/mL)	IGF-1 (pg/mL)
Ad-mIGF-1 group	3.636±0.509	355.57±15.81	45.54±3.10	147.07±10.36	8.17±0.36
Ad-mIL-10 group	4.491±0.338	295.62±20.29	45.06±1.48	183.02±11.40	6.87±0.67
Combined intervention group	5.153±0.262	265.02±12.40	57.84±2.39	210.58±8.27	8.53±0.49
Diabetic control group	3.296±0.328	476.72±27.12	34.58±2.40	124.95±9.49	3.93±0.44
Control group	7.050±0.500	234.42±28.44	78.72±5.89	255.96±31.10	11.52±1.03

### Degree of Insulitis in Mice

**Figure 2:**
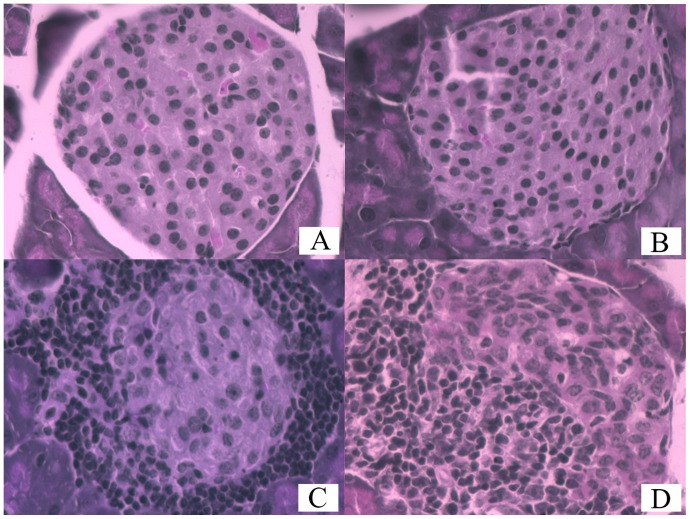
The degree of inflammation infiltration of islet cells. A, B, C and D represent Grade 0, 1, 2 and 3 of inflammation infiltration of islet cells in HE stained section under microscope (x400).

**Figure 3:**
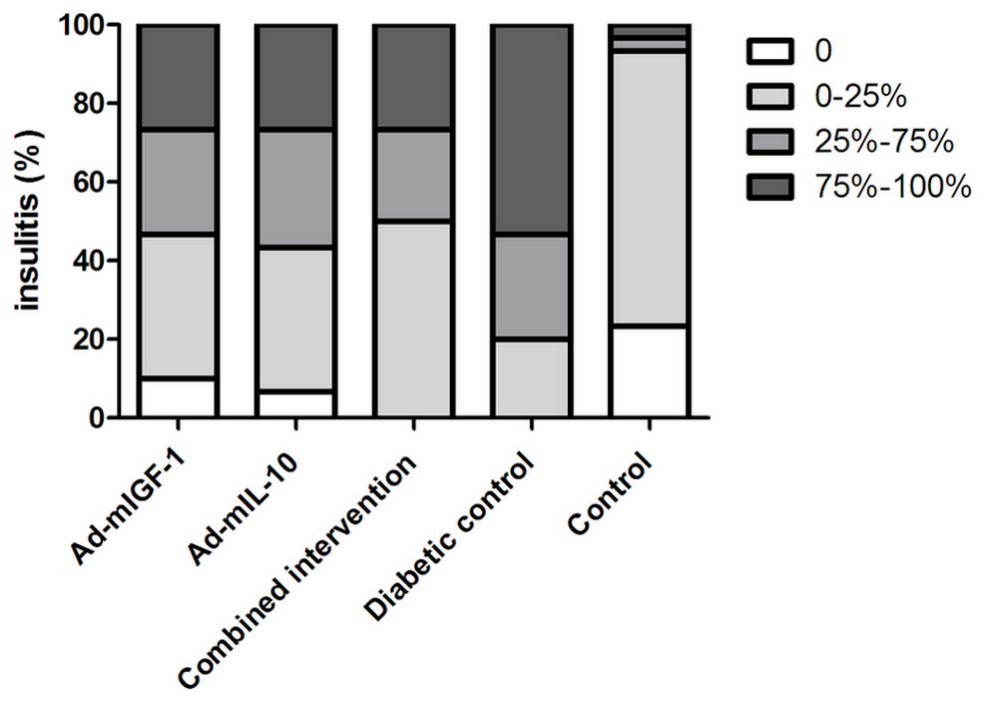
Ad-m IL-10/Ad-m IGF-1 combined treatment affects insulitis in NOD mice. Mice were treated with Ad-m IL-10/Ad-m IGF-1. Pancreata were collected at 3 weeks after treatment, and insulitis was scored as follows: 0 = no insulitis, 1 = <25% infiltration, 2 = 25–75% infiltration, and 3 = >75% infiltration. Insulitis was scored in 30 islets. *P<*0.05 for groups 1 and 4 vs control group.

The degree of insulitis was examined by microscopy after HE staining (Figure 2 and 3) and graded based on the criteria described above. As shown in Table 3, the degree of insulitis of mice in group 5 was graded as 0 or 1, while that of mice in other groups was graded as 1, 2 or 3. In particular, insulitis grade of mice in group 4 was 3, showing smaller and fewer islets, obvious infiltration of inflammatory cells (mainly lymphocytes in islet and around islets), and abnormal islet cell morphology (Figure 2-D). Kruskal-Wallis rank sum test and pairwise comparison test at level of α’ = α/10 = 0.05/10: 10 = 0.005 indicated that the degree of infiltration was significantly higher in mice in groups 1, 2, 3 and 4 than in mice in group 5 (*P*<0.05), but that was not significantly different among mice in groups 1, 2, 3, and 4 (*P*>0.05).

**Table 3:** The number of islets in mice with insulitis at different grades.

Groups	Grade 0	Grade 1	Grade 2	Grade 3
Ad-mIGF-1 group	3	11	8	8
Ad-mIL-10 group	2	11	9	8
Combined intervention group	0	15	7	8
Diabetic control group	0	6	8	16
Control group	7	21	1	1

Notes: *P<*0.05 for groups 1 and 4 vs control group.

### Expression of IGF-1 and IL-10 in Pancreas

**Figure 4:**
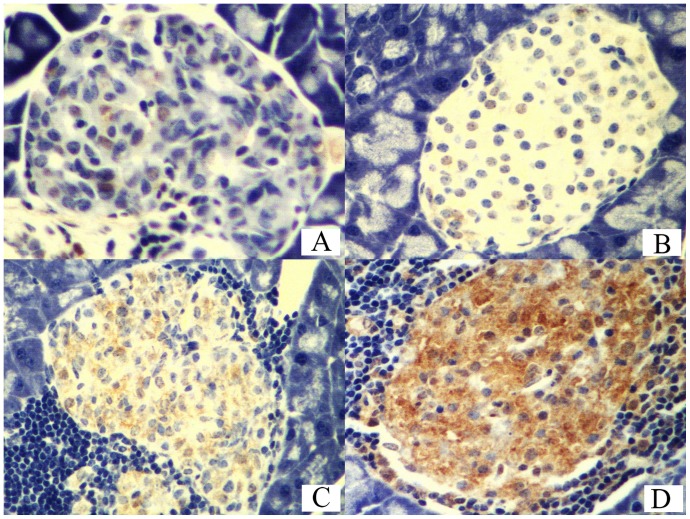
Immunohistochemical staining Level of pancreatic biopsy. A, B, C and D represent grade (−), (+), (++) and (+++) under microscope, respectively (x400).

The level of IGF-1 and IL-10 in pancreas was examined using immunohistochemical SABC method. As shown in Table 4 and Figure 4, the expression level of IL-10 of the mouse 2 and 3 groups was significantly higher than that 1 and 4 groups (*P*<0.01). In addition, the mouse group 1 level of IL-10 was significantly lower than that 2 and 3 groups (*P*<0.01), and there was no significant difference in IL-10 level between mouse 3 and 5 groups (*P*>0.05). Compared with the mouse 1 and 3 groups and 2 and 4 groups, we found significantly more IGF-1 in pancreas (*P*<0.01), but significantly lower than that the mouse of 5 group (*P*<0.01). In addition, IGF level of the mouse 2 group was significantly higher than that 4 group (*P*<0.01).

**Table 4:** Immunostaining score of IGF-1 and IL-10 of mice in different groups (x¯±s).

Groups	No. of Cases	IGF-1	IL-10
Ad-mIGF-1 group	6	5.67±0.82^▴^	2.33±1.37
Ad-mIL-10 group	6	2.50±1.22	5.62±0.81^▾^
Combined intervention group	6	5.00±1.10^▴^	5.02±1.09^▾^
Diabetic control group	6	1.59±0.55	1.50±0.54
Control group	6	4.33±0.82^▴^	4.67±1.03^▾^

Notes: *P* ^▴^<0.05 for IGF-1score vs that of mice in group 4; *P* ^▾^<0.05 for IL-10 score vs that of mice in group 4.

## Discussion

Over the years, people have been committed to study T1D pathogenesis and prevention methods and gradually realized that the ultimate goal for function reconstruction of endogenous islet β-cells is to find interventions such as antigen-specific ones (insulin, glutamic acid decarboxylase), antibody-dependent ones (anti-CD3 and anti-CD20 monoclonal antibodies), immunosuppressive agents (cyclosporin and azathioprine), cytokines (IL-10, IGF-1), etc, to protect islet β-cells. But the outcomes of clinical applications of these interventions are not satisfactory, mainly due to low maximum dosage caused by side effects and the complexity of the pathogenesis of T1D [10], [11], [12], [13]. According to the characteristics of T1D at different stages, selecting appropriate combined application could, on the one hand, reduce the toxic side effects, on the other hand, achieve synergistic and complementary effects at the aspects of pathways and mechanisms. Based on this theory, we explored the effects of application of both intervention reagents IL-10 and IGF-1, and observed their protective effects on pancreatic β-cells of NOD mice at onset of T1D.

IGF-1 is to regulate glucose metabolism and other biological effects by binding to its receptor and acts as a growth factor to regulate cell growth, survival and metabolism, C-peptide secretion, insulin sensitivity and immune responses. It has been discovered in recent years that IGF-1 also regulates the regeneration of pancreatic β-cells, thus is closely related to T1D [32], [33].

The immune imbalance theory is generally believed that the imbalance of Th1/Th2 cell subsets and immune dysfunction are the pathogenic causes of T1D [34]. Th2 and their cytokine products, particularly IL-10, an important immune regulatory factor, have strong inhibitory effects on autoimmune inflammatory process, antigen presentation, inflammatory cell activation, cytokine secretion and others [35]. IL-10 can not only inhibit the expression of TNF-α and IL-1, and the generation of free radical oxygen products, but also the release of IFN-γ through inhibiting IL-2 production of the antigen-presenting cells [36], [37]. Therefore, application of IL-10 has an important role in rebalance of Th1/Th2 cell subsets.

Since the half-life of exogenous IL-10 and IGF-1 is relatively short, they have to be constantly and repeatedly administrated. Moreover, systemic administration of drugs has poor target, limiting its clinical application. Transgenic technology could introduce the targeted genes into targeted cells and highly express these genes locally, thus maintaining a high local drug concentration in a longer duration [16]. In this experiment, adenovirus-mediated IL-10 and IGF-1 genes were successfully introduced into mice in vivo. Their level was very high in both pancreas and serum. In addition, expression of IL-10 and IGF-1 showed no significant side effects, indicating they played a good intervention role.

When NOD mice had insulitis, they gradually showed phenomena such as hyperglycemia, polyphagia, polydipsia, polyuria, irritability and decreased hair gloss [3]. In the NOD mouse, the progresses of insulitis are peri-insulitis at 3–4 weeks, invading insulitis at 8–10 weeks and destrucive insulitis at 14–16 weeks of age [11]. Three weeks after injecting IL-10 or IL-10 plus IGF-1, their body weight showed a downward trend, and was significantly lower than that of mice in the control group and in IGF-1 treatment group. Moreover, dynamic observation of blood glucose level found that mice treated with either IL-10 or IGF-1 had similar blood glucose level with diabetic control mice, while mice treated with IL-10 plus IGF-1 had lower blood glucose level than mice treated with either IL-10 or IGF-1 and diabetic control mice and higher glucose level than mice in the control group, but these differences were not statistically significant. Similarly, mice treated with IL-10 plus IGF-1 also had slightly attenuated polyphagia, polydipsia, polyuria, irritability, lower blood glucose level and body weight, although not statistically significant. Meanwhile, in this study, compared with mice in diabetic control group, the islet inflammation degree of mice in genetic intervention group was only slightly reduced (*P*>0.05), indicating that IL-10 and/or IGF-1 gene intervention could not alleviate islet inflammation. Maybe it is related to the large number of regulatory cells within the pancreatic lesion.

It is generally agreed that imbalances between Th1/Th2 cell subsets and among their cytokines are the key pathogenic causes for T1D. The study showed that mice treated with IL-10 or IGF-1, especially mice treated with IL-10 plus IGF-1 had significantly lower serum Th1-type cytokines such as IFN-γ and higher serum Th2-type cytokines such as IL-4 and IL-10 than mice without treatment (*P*<0.01), indicating that gene intervention played certain roles in rebalancing Th1/Th2 subsets. Compare with mice treated with IGF-1, mice treated with IL-10 had significantly reduced IFN-γ level and enhanced IL-10 level (*P*<0.01), suggesting that IL-10 had better intervention outcomes. Compared with mice in the control group, all diabetic mice had significantly elevated IFN-γ level and decreased IL-4 level (*P*<0.01), supporting the theory of Th1/Th2 cell subsets imbalance and confirming that gene intervention with IGF-1 and/or IL-10 played certain roles in rebalancing Th1/Th2 subsets, but could not completely reverse the imbalance. Numerous strategies have been reported to either delay or prevent T1D in NOD mice [11], [12], [13]. However, very few therapies have been demonstrated to either reverse new-onset T1D in NOD mice. For our research, the reasons maybe as follows: first, the progression of T1D is very complex and many regulatory cells such as inducible costimulator molecule(ICOS), CD154 or CTLA-4Ig and so on are relevent to the apoptosis of β-cells [11], [12], [13]. Only reduced IFN-γ and enhanced IL-10 level could not reverse the insulitis. Second, the beginning of treatment maybe should at prediabetic pieriod and the duration of treatment should be longer. Wen Li et al. reported combined treatment with intracenous antihuman CD20(hCD20) and oral anti-CD3 could delayed diabetes development in prediabetic hCD20 transgenic NOD mice and reversed diabetes in >60% of mice newly diagnosed with diabetes. Further mechanistic studies showed that the combined therapy enhanced the suppressive function of regulatory T-cells, IL-10- and IL-27-producing dendritic cells. They demonstrated that there was a significant reduction of insulitis 1 month after treatment, but at 15 days and 3 months posttreatment, there were no significant differences in cellular infitration in mice treated with anti-hCD20/oral anti-CD3 compared with those treated with control IgGs [10]. Another recent paper also showed that anti-CD22 immunotherapy can deplete and reprogram B-cells, therapy reversing autoimmne diabetes in naïve NOD mice [38].

C-peptide is a precursor of insulin. Its secretion level directly reflects the functions of pancreatic β-cells. In this study, we determined serum C-peptide level using ELISA and found that C-peptide level of mice in combined gene intervention group was significantly elevated compared with that of mice in single gene intervention group and in diabetic control group, but still lower than that of mice in the control group. In addition, C-peptide level of mice in IL-10 intervention group, but not IGF-1 intervention group, was higher than that of mice in diabetic group, suggesting that IL-10, but IGF-1, could protect pancreatic β-cells and IGF-1 could enhance the effects of IL-10 gene.

In summary, at the onset of T1DM, adenovirus-mediated IL-10 and/or IGF-1 intervention enhanced expression of IL-10 and/or IGF-1 in pancreas and serum, which in turn increased the expression of protective Th2-type cytokines (IL-4, IL-10) in pancreatic β-cells, reduced the level of destructive Th1-type cytokines (IFN-γ) in pancreatic β-cells, and increased serum C-peptide level, thus exerting their protective function and rebalancing Th1/Th2 subset cells in pancreatic β-cells. However, IL-10 and/or IGF-1 intervention could not improve blood glucose and body mass and reduce insulitis. This is possibly because that after the onset of T1D, the inflammatory infiltration of islets was very severe and most islet β-cells had been damaged. Although genetic intervention had certain immunomodulatory and protective roles to islet cells, it could not completely prevent insulitis process, improve the clinical manifestations of diabetes such as enhanced blood glucose and body weight. The study suggested that effects of “cocktail therapy” of combined genetic intervention of IL-10 and IGF-1 were not satisfactory and other more effective cocktail therapies need to be further explored.
